# HIV-1 drug-resistant mutations and related risk factors among HIV-1-positive individuals experiencing treatment failure in Hebei Province, China

**DOI:** 10.1186/s12981-017-0133-3

**Published:** 2017-01-23

**Authors:** Xinli Lu, Hongru Zhao, Yuqi Zhang, Wei Wang, Cuiying Zhao, Yan Li, Lin Ma, Ze Cui, Suliang Chen

**Affiliations:** Hebei Provincial Center for Disease Control and Prevention, 97 Huaian East Road, Yuhua District, Shijiazhuang, 050021 China

**Keywords:** HIV-1, Mutation, Phylogeny, Drug resistance, China

## Abstract

**Background:**

To understand HIV-1 drug resistance in 11 prefectures of Hebei Province, China, we implemented a cross-sectional HIV-1 molecular epidemiological survey.

**Methods:**

Blood samples were collected from 122 newly diagnosed drug-naïve HIV-1-positive individuals and 229 antiretroviral therapy (ART)-failure individuals from 11 prefectures in Hebei Province, China. Patient demographic data were obtained via face-to-face interviews using a standardized questionnaire when blood samples were collected. Genotyping of HIV-1 drug resistance (DR) was implemented using an in-house assay.

**Results:**

In this study, the overall prevalence of HIV-1 DR was 35.5%. The prevalence of HIV-1 DR in participants experiencing treatment failure and ART-naïve participants was 51.9 and 5.9%, respectively. Mutations in protease inhibitors, nucleoside reverse transcriptase inhibitors (NRTIs), and non-NRTI (NNRTIs), as well as dual and multiple mutations were extensively seen in participants experiencing treatment failure. The proportions of NNRTI mutations (χ^2^ = 9.689, *p* = 0.002) and dual mutations in NRTIs and NNRTIs (χ^2^ = 39.958, *p* < 0.001) in participants experiencing treatment failure were significantly higher than those in ART-naïve participants. The distributions of M184V/I and M41L mutations differed significantly among three main HIV-1 genotypes identified. Viral load, symptoms in the past 3 months, CD4 counts, transmission route, and the duration of ART were found to be associated with HIV-1 DR.

**Conclusions:**

Our results suggest that new prevention and control strategies should be formulated according to the epidemic characteristics of HIV-1-resistant strains in Hebei Province, where antiretroviral drugs are widely used.

**Electronic supplementary material:**

The online version of this article (doi:10.1186/s12981-017-0133-3) contains supplementary material, which is available to authorized users.

## Background

Human immunodeficiency virus (HIV) epidemics can be traced back to the 1920s in Kinshasa, the capital of the Democratic Republic of the Congo [1]. Among the first HIV-1 individuals in China were four hemophiliac patients in 1985 [2]. Some early cases of HIV infection were linked to imported blood products [3]. In 1989, an HIV outbreak occurred among injection drug users (IDUs) in Yunnan Province, China [4]. Since then, individuals with HIV or AIDS have been successively identified in provinces of mainland China [5–7], and an estimated 740,000 individuals in China are currently thought to be infected with HIV/AIDS [8]. Over the past 30 years, the most common route of transmission of HIV-1 infection in China has shifted from blood products to sexual contact [9], and the genetic diversity has rapidly increased because of HIV-1 gene hypermutability [10].

Hebei Province, China comprises 11 prefectures, surrounds the cities of Beijing and Tianjin, and neighbors Henan Province to the south. In 2014, it was inhabited by more than 73 million people [11]. The first case of HIV infection in Hebei Province was detected in Shijiazhuang in 1989 [12]. In the 1990s, local HIV outbreaks occurred in Xingtai and Langfang, and many individuals infected with HIV-1 through blood transmission were identified [13, 14]. More recently, HIV infection has been detected in all 172 counties of Hebei Province, and sexual exposure, especially in men-who-have-sex-with-men population, has gradually replaced blood transmission as the most common transmission route [15]. By the end of 2014, a total of 5315 HIV/AIDS cases had been reported, including 3050 HIV-1-positive individuals and 2265 AIDS patients. The HIV/AIDS infection rate in Hebei was estimated to be 0.011%, which is significantly lower than the 0.059% reported for the whole of China and the 0.8% worldwide [16], representing a low HIV/AIDS epidemic.

Before 2002, it was not practical to use antiretroviral therapy (ART) in China due to a lack of drug access, and HIV-1 drug-resistant strains were rare [17]. Since 2003, the central government has provided free ART to HIV/AIDS patients, and first-line regimens are commonly used in Hebei. By the end of October 2014, 167 of 172 counties in Hebei had carried out the “four free, one care” policy [18], and a total of 2893 HIV/AIDS patients received highly active ART. This represented a large increase in ART coverage, from 9.9% in 2003 to 96.6% in 2014, which coincided with a significant decrease in HIV/AIDS patient mortality from 11.6 to 2.6% [16]. However, with the increase in antiretroviral drug use, the frequency of adaptive mutations in HIV-1 has also increased, generating drug-resistant strains [19]. This has created severe clinical and epidemiological problems [20].

The objective of the present study was to perform a detailed analysis of the prevalence and genetic mechanisms of HIV-1 drug resistance (DR) among participants experiencing treatment failure in Hebei, and to evaluate the underlying influencing factors associated with the development of HIV-1 drug-resistant strains.

## Methods

### Participants

**Fig. 1:**
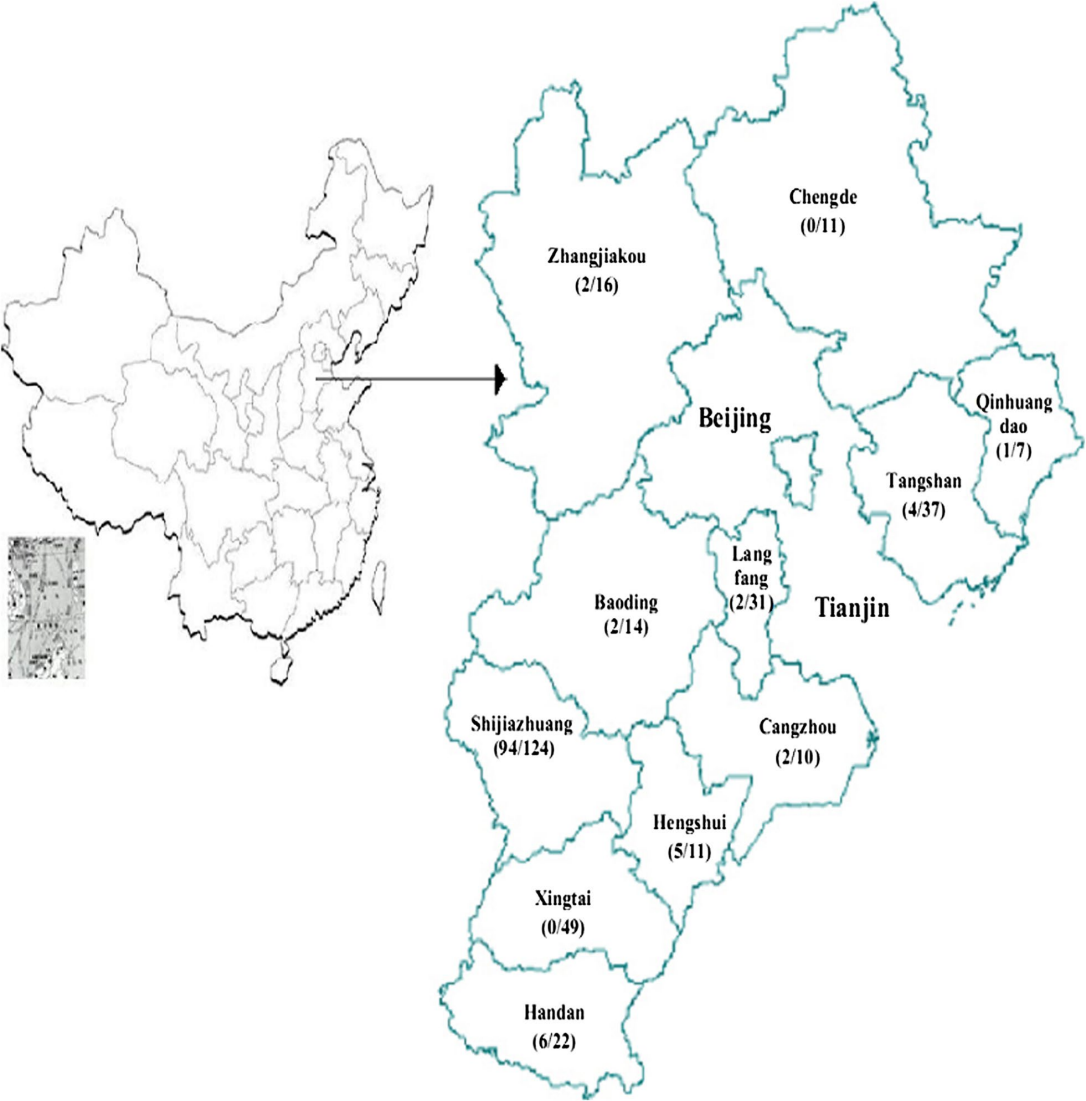
Geographical distribution of participants collected from 11 prefectures in this study. The *numbers* to the *left* and *right* of the “/” denote the participants genotyped and the total participants, respectively. This figure is adapted from open access map: http://wenku.baidu.com/link?url=u_b5Oe5nC1s_dm7nivfQ1VxQcwj9lDMPsoWfHZHGUNJM5IUiv7JnZo1yAWlVx9KbITt2u5tReJ7qPOtnoxeJw3QI1VUewd1m9N56eJSxuHm and figure 1 in Ref. [14] with Microsoft PowerPoint 2016

Between October 2012 and April 2013, 351 whole blood samples were collected from 122 newly diagnosed drug-naïve HIV-1-positive individuals confirmed in 2012 and 229 participants experiencing treatment failure in 11 prefectures of Hebei (Fig. 1). We selected participants experiencing treatment failure according to the following criteria: (1) viral load (VL) ≥1000 copies/ml, (2) duration of therapy >6 months, (3) CD4 count lower than the level before ART, and (4) genotyping had not been previously performed. The local centers for disease control and prevention were responsible for the delivery of antiretroviral drugs and sample collection. Controls were 122 newly diagnosed HIV-1-positive individuals who had not received treatment. The study design was cross-sectional.

Demographic data were collected via face-to-face interviews when blood samples were collected, using a standardized questionnaire. A total of 50 µl of whole blood was used to measure the CD4 count using a FACSCount reagent kit (Becton–Dickinson, Franklin Lakes, NJ, USA). Plasma samples were obtained by centrifuging whole blood, and used to detect VL with the COBAS TaqMan 48 analyzer (Roche, Basel, Switzerland).

### HIV-1 genotyping and drug resistance

HIV-1 RNA was extracted from 500 µl of blood plasma using the High Pure Viral RNA kit (Qiagen, Valencia, CA, USA). The partial HIV-1 *pol* gene fragment (HXB2:2147–3462) was amplified for HIV-1 genotyping and DR using the One-Step reverse transcription PCR kits (TaKaRa, Dalian, China) with primers MAW26 (5′-TTGGAAATGTGGAAAGGAAGGAC-3′) and RT21 (5′-CTGTATTTCTGCTATTAAGTCTTTTGATGGG-3′) in a 25 µl reaction volume. Cycling conditions were as follows: HIV-1 RNA denaturation at 65 °C for 30 s, addition of the reaction mixtures at 4 °C, incubation at 50 °C for 30 min, 94 °C for 2 min, then 35 cycles of 94 °C for 30 s, 55 °C for 30 s, and 72 °C for 2 min 30 s.

Nested *pol* PCR was implemented using 2× Taq PCR MasterMix (TaKaRa) with primers PRO-1 (5′-CAGAGCCAACAGCCCCACCA-3′) and RT20 (5′-CTGCCAGTTCTAGCTCTGCTTC-3′) in a 50 µl reaction volume. Cycling conditions were: 94 °C for 5 min, then 35 cycles of 94 °C for 30 s, 63 °C for 30 s, and 72 °C for 2 min 30 s. Positive PCR products were analyzed using 1% agarose gel electrophoresis, and sequenced by Biomed (Beijing, China).

All original *pol* sequence fragments were assembled, edited, and aligned as previously described [21], and used to construct an HIV-1 *pol* phylogenetic tree using the neighbor-joining method with 1000 bootstrap replicates, based on the Kimura 2-parameter Model (MEGA5.0). The online jpHMM Program (http://jphmm.gobics.de/submission_hiv.html) and RIP 3.0 (http://www.hiv.lanl.gov/content/sequence/RIP/RIP.html) were used to further analyze the possible intertype mosaicism of unique recombinant forms (URFs). Finally, HIV-1 *pol* sequences were submitted to the HIV DR database (http://hivdb.stanford.edu/) to analyze HIV-1 DR mutations.

### Statistical analysis

Statistical analyses were implemented using SPSS software version 21.0 (SPSS Inc., Chicago, IL, USA). Means or frequencies of demographic data (such as age, CD4 counts, and VL) were calculated. Categorical variables were analyzed using the Chi square test. When more than 20% of cells had an expected count of <5, Fisher’s exact test was used. Multivariable logistic regression analysis was used to identify risk factors associated with DR. A stepwise approach was used for variable selection in the multivariate regression model. All tests were two-sided, and a statistical result was considered significant when *p* < 0.05.

## Results

### Demographic characteristics of participants

Table 1 shows the demographic characteristics of participants. The sex ratio of males to females was 1:0.27. The median values of age, CD4 counts, and VL were 37.0 (range 6–71) years, 220 (range 2–1149) cells/μl, and 4.2 (range 3–6.8) log RNA copies/ml, respectively. Sexual contact was the most common transmission route in the study participants and accounted for 76.1% of transmission (267/351), including heterosexual contact (28.8%, 101/351) and homosexual contact (47.3%, 166/351), followed by blood (17.9%, 63/351), mother-to-child transmission (MTCT, 5.4%), and IDU (0.6%). In terms of ethnicity, 98.0% (344/351) of participants were Chinese Han, and the remaining seven participants were Hui (0.9%, 3/351), Yi (0.7%, 2/351), Man (0.4%, 2/351), and Uyghur (0.4%, 1/351).

**Table 1:** Demographic characteristics of participants in this study

Characteristic	Individuals (%)		
	ART-naïve (n = 122)	ART-failure (n = 229)	Cumulative (n = 351)
Age, median years (IQR)	31.5 (17–71)	39.5 (6–71)	37.0 (6–71)
Gender			
Male	116 (95.1)	160 (69.9)	276 (78.6)
Female	6 (4.9)	69 (30.1)	75 (21.4)
Median CD4+ T cell count, cells/μL (IQR)	432.50 (9–1149)	188 (2–556)	220 (2–1149)
Median VL, RNA (lgcopies/ml) (IQR)	4.6 (3.2–6.8)	4.2 (3.0–6.5)	4.22 (3.0–6.8)
Ethnicity			
Han	121 (99.2)	223 (97.4)	344 (98.0)
Hui	1 (0.8)	2 (0.9)	3 (0.9)
Yi	0 (0.0)	2 (0.9)	2 (0.7)
Uyghur	0 (0.0)	1 (0.4)	1 (0.4)
Man	0 (0.0)	1 (0.4)	1 (0.4)
Transmission routes			
Heterosexual	23 (1.9)	78 (34.1)	101 (28.8)
Homosexual	99 (98.1)	67 (29.3)	166 (47.3)
MTCT	0 (0.0)	19 (8.3)	19 (5.4)
Blood recipient	0 (0.0)	34 (14.9)	34 (9.7)
Paid blood donor	0 (0.0)	29 (12.7)	29 (8.3)
IDU	0 (0.0)	2 (0.9)	2 (0.6)

*IQR* interquartile range, *IDU* intravenous drug injection, *MTCT* mother-to-child transmission

**Fig. 2:**
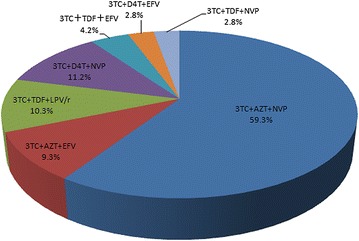
The therapy regimens of ART participants in this study. *ATV/r* atazanavir/r, *NFV/r* nelfinavir plus ritonavir, *LPV/r* lopinavir plus ritonavir, *3TC* lamivudine, *ABC* abacavir, *AZT* zidovudine, *D4T* stavudine, *DDI* didanosine, *FTC* emtricitabine, *TDF* tenofovir, *EFV* efavirenz, *ETR* etravirine, *NVP* nevirapine

Among all therapy regimens in 214 participants experiencing treatment failure (Fig. 2), the 3TC + AZT + NVP regimen was the most frequent, accounting for 59.3%. The percentage of participants treated with 3TC + D4T + NVP, 3TC + TDF + LPV/r, 3TC + AZT + EFV, 3TC + TDF + EFV, 3TC + D4T + EFV, and 3TC + TDF + NVP was 11.2, 10.3, 9.3, 4.2, 2.8 and 2.8%, respectively.

### HIV-1 genotype analysis

Viral RNA isolated from 332 out of 351 participants was amplified and sequenced successfully, including 118 from ART-naïve controls (96.7%, 118/122) and 214 from participants experiencing treatment failure (93.4%, 214/229), achieving a positive sequence rate of 94.6% (332/351). As shown in Additional file 1: Table S1 and Figures S1, S2, seven HIV-1 genotypes were identified successfully by the phylogenetic tree analyses of HIV-1 *pol* sequences. HIV-1 subtype B (41.9%, 139/332) was identified as the most frequent genotype, followed by circulating recombinant form (CRF)01_AE (40.1%, 133/332), CRF07_BC (13.6%, 45/332), CRF08_BC (2.1%, 7/332), subtype C (1.2%, 4/332), URFs (0.6%, 2/332), and CRF02_AG (0.6%, 2/332). We identified two URF recombination patterns: CRF01_AE/BC and CRF01_AE/B (Additional file 1: Figure S3). This is the first known identification of CRF02_AG in Hebei.

The prevalent genotypes in the present work were identical to those detected in our previous study [21], with the exception of CRF02_AG. Furthermore, the HIV-1 genotype distribution in 11 prefectures (Additional file 1: Figure S4) was associated with changes of transmission routes (Additional file 1: Table S2), consistent with the geographical distribution characteristics of HIV-1 genotypes reported previously [21]. This suggests that the geographical difference of transmission routes plays a critical role in this distribution.

### HIV-1 drug-resistant mutations in ART-naïve controls

In ART-naïve controls, the prevalence of HIV-1 DR was 5.9% (7/118), including protease inhibitor (PI) mutations (2.5%, 3/118), nucleoside reverse transcriptase inhibitor (NRTI) mutations (1.7%, 2/118), non-NRTI (NNRTI) mutations (0.8%, 1/118), and dual mutations in NRTIs and NNRTIs (0.8%, 1/118). One participant infected through heterosexual contact harbored dual mutations in NRTIs (T69N, M184V, and T215Y) and NNRTIs (K103N and M230L), and presented with high-level resistance to lamivudine (3TC) and emtricitabine (FTC) with M184V, intermediate-level resistance to zidovudine (AZT) and stavudine (D4T) with T215Y, and intermediate or high-level resistance to all NNRTIs with K103N and M230L. Two participants harboring M46L and one with M46 V showed low-level resistance to nelfinavir plus ritonavir (NFV/r). Two participants infected through homosexual contact and one participant infected through heterosexual contact harbored NRTI mutations D67N and M184V, and NNRTI mutation K103N. The proportions of resistance to NFV/r, 3TC, FTC, AZT, D4T, EFV, ETR, NVP, and RPV were 2.5% (3/118), 1.7% (2/118), 1.7% (2/118), 1.7% (2/118), 1.7% (2/118), 1.7% (2/118), 0.8% (1/118), 1.7% (2/118), and 0.8% (1/118), respectively.

### HIV-1 drug-resistant mutations in participants experiencing treatment failure

Compared with the low prevalence of HIV-1 DR in ART-naïve controls, 51.9% (111/214) of participants experiencing treatment failure showed resistance to at least one antiviral drug. Mutations in PIs, NRTIs, and NNRTIs, and dual and multiple mutations were common in participants experiencing treatment failure. As shown in Table 2, the mutation classes showed significant differences in frequency between ART-naïve participants and participants experiencing treatment failure (*p* = 0.014). The proportions of NNRTIs mutations (χ^2^ = 9.689, *p* = 0.002) and dual mutations in NRTIs and NNRTIs (χ^2^ = 39.958, *p* < 0.001) in participants experiencing treatment failure were significantly higher than those in ART-naïve participants. Furthermore, dual mutations in NRTIs and NNRTIs were the most common mutation class in participants experiencing treatment failure, accounting for 29.4% (63/214), followed by NNRTI mutations (10.7%, 23/214).

**Table 2:** Drug resistance in ART-Naïve participants and participants experiencing treatment failure according to drug classes

Drug classes	ART-naïve (%)	ART-failure (%)	χ^2^	p
PIs	3 (2.5)	6 (2.8)	0.000	1.000
NRTIs	2 (1.7)	9 (4.2)	0.816	0.366
NNRTIs	1 (0.8)	23 (10.7)	9.689	0.002
Dual resistance to PIs and NRTIs	0 (0.00)	1 (0.5)	F	1.000
Dual resistance to PIs and NNRTIs	0 (0.00)	6 (2.8)	F	0.093
Dual resistance to NRTIs and NNRTIs	1 (0.8)	63 (29.4)	39.958	<0.001
Multiple resistance to PIs, NRTIs and NNRTIs	0 (0.00)	3 (1.4)	F	0.555
Susceptibility	101 (85.6)	103 (48.1)	45.059	<0.001

*PIs* protease inhibitors, *NRTIs* nucleoside reverse transcriptase inhibitors, *NNRTIs* non-nucleoside reverse transcriptase inhibitors, the mutation classes were exclusive of each other in the study population, *F* Fisher’s exact test

Table 3 lists all mutations that cause different levels of DR to antiviral drugs. In PI coding regions, mutations T74S and M46L were found to cause low-level DR to NFV/r, achieving a resistance rate of 1.8% (4/214). In NRTI coding regions, M184V/I was the most frequent mutation, accounting for 30.4% (65/214), followed by K70R (8.4%, 18/214), D67N (5.6%, 12/214), M41L (5.4%, 11/214), and T215Y (5.4%, 11/214). The percentages of resistance to 3TC, ABC, FTC, AZT, D4T, DDI, and TDF were 30.4% (65/214), 28.5% (61/214), 30.4% (65/214), 22.9% (49/214), 24.3% (52/214), 12.1% (26/214), and 9.8% (21/214), respectively. In NNRTI coding regions, K103 N was the most frequent mutation, accounting for 15.9% (34/214), followed by Y181C (11.7%, 25/214), G190A (5.1%, 11/214), and G190S (3.7%, 8/214). The percentages of resistance to EFV, ETR, NVP, and RPV were 37.4% (80/214), 21.5% (46/214), 37.4% (80/214), and 23.8% (51/214), respectively. The overall prevalence of HIV-1 DR was 35.5% (118/332).

**Table 3:** Prevalence of drug-resistance mutations among ART-naïve participants (n = 118) and participants experiencing treatment failure (n = 214) in Hebei between 2012 and 2013

Mutations	Frequency (%)		HIV-1 drug resistance level							
	Naïve (n = 118)	ART-failure (n = 214)								
			NFV/r	*ATV/r*	*DRV/r*	FPV/r	IDV/r	*LPV/r*	SQV/r	TPV/r
Protease inhibitors										
L10V	0.85	1.40	S	S	S	S	S	S	S	S
L10I	4.24	0.47	S	S	S	S	S	S	S	S
T74S	0.00	0.93	L	S	S	S	S	S	S	S
A71V/T	11.86	2.80	S	S	S	S	S	S	S	S
K20I	0.85	0.93	P	S	S	S	S	S	S	S
L33I	0.85	1.40	S	S	S	S	S	S	S	S
M46L	1.69	0.93	L	P	S	P	P	P	S	S
M46V	0.85	0.00	L	P	S	P	P	S	S	S
G48W	0.85	0.00	S	S	S	S	S	S	S	S
V11I	0.85	0.00	S	S	S	S	S	S	S	S
K43T	0.85	0.00	S	S	S	S	S	S	S	P

			*3TC*	*ABC*	*AZT*	*D4T*	*DDI*	*FTC*	*TDF*	
Nucleoside reverse transcriptase inhibitors										
M184V	1.69	27.57	H	L	S	S	P	H	S	
Q151M	0.00	0.47	L	H	H	H	H	L	L	
F116Y	0.00	0.47	S	P	P	P	P	S	S	
M184I	0.00	1.87	H	P	S	S	S	H	S	
T69N	0.85	2.34	S	S	S	S	P	S	S	
T69i	0.00	0.47	M	M	M	M	M	M	M	
A62V	0.00	1.87	S	S	S	S	S	S	S	
M41L	0.00	5.14	S	P	L	L	P	S	P	
D67S	0.00	0.47	S	S	P	P	S	S	S	
V75I	0.85	0.47	S	S	S	P	P	S	S	
V75L	0.00	2.80	S	S	S	P	P	S	S	
T215I	0.00	0.93	S	P	L	L	P	S	S	
T215Y	0.85	5.14	S	L	M	M	L	S	L	
K70T	0.00	0.47	P	P	S	P	P	P	P	
K70E	0.00	0.47	P	L	S	S	L	P	L	
K70R	0.00	8.41	S	P	M	L	P	S	P	
V75M	0.00	0.93	S	S	P	M	L	S	S	
L210W	0.00	0.93	S	P	L	L	P	S	P	
D67N	0.85	5.61	S	S	L	L	S	S	S	
D67G	0.00	0.47	S	S	P	P	S	S	S	
T215F	0.00	2.34	S	L	M	M	L	S	L	
T215N	0.00	0.47	S	P	L	L	P	S	S	
K219E	0.00	3.27	S	S	P	P	S	S	S	
K219Q	0.00	2.34	S	S	P	P	S	S	S	
L74I	0.00	1.40	S	L	S	S	H	S	S	
L74V	0.00	0.93	S	M	S	S	H	S	S	
K65R	0.00	1.87	M	M	S	M	H	M	H	

			*EFV*	*ETR*	*NVP*	RPV				
Non-nucleoside reverse transcriptase inhibitors										
A98G	0.00	1.40	P	P	M	L				
H221Y	0.00	6.54	P	P	P	P				
K101E	0.00	3.27	L	L	M	M				
K103N	1.69	15.89	H	S	H	S				
K103S	0.00	0.47	M	S	H	S				
K103T	0.00	0.47	L	S	H	S				
V106A	0.00	0.93	M	P	H	S				
V106M	0.00	1.40	H	S	H	S				
V106I	7.63	8.88	S	S	S	S				
V108I	0.00	3.74	P	S	L	S				
E138Q	0.00	2.34	P	P	P	L				
E138G	0.85	0.0	P	P	P	L				
V179E	4.24	2.80	P	P	P	P				
V179D	0.85	0.47	P	P	P	P				
Y181C	0.00	11.68	M	M	H	M				
Y188L	0.00	1.40	H	L	H	H				
Y188C	0.00	0.47	M	S	H	S				
Y188W	0.00	0.47	S	S	S	S				
G190S	0.00	3.74	H	L	H	L				
G190A	0.00	5.14	M	L	H	L				
G190R	0.00	0.47	S	S	S	S				
P225H	0.00	1.87	M	S	L	S				
F227L	0.00	0.93	L	S	M	S				
M230L	0.85	0.47	M	M	H	M				
M230I	0.00	0.93	L	L	M	M				
K238T	0.00	2.34	L	S	M	S				
V90I	0.00	5.14	S	S	S	S				
E138A	0.00	0.93	S	P	S	L				
E138K	0.00	0.47	P	P	P	M				

Some (italics) of drugs listed in the Stanford HIV DR database are used in China

*S* susceptible, *P* potential low-level resistance, *L* low-level resistance, *M* intermediate resistance, *H* high-level resistance, *ATV/r* atazanavir/r, *NFV/r* nelfinavir plus ritonavir, *LPV/r* lopinavir plus ritonavir, *3TC* lamivudine, *ABC* abacavir, *AZT* zidovudine, *D4T* stavudine, *DDI* didanosine, *FTC* emtricitabine, *TDF* tenofovir, *EFV* efavirenz, *ETR* etravirine, *NVP* nevirapine

Additionally, 5.1% (11/214) of participants experiencing treatment failure harbored thymidine analogue mutations (TAMs; Table 4), accounting for 9.9% (11/111) of participants identified as DR. As shown in Table 4, the mean therapeutic duration of the 11 participants with TAMs was 42.8 (range 10–113) months, the mean VL was 4.3 (range 3.2–5.3) log copies/ml, and the mean CD4 count was 108.9 (range 7–187) cells/μl. Sexual transmission accounted for 90.9% (10/11) of cases, with heterosexual transmission accounting for 81.8% (9/11). TAMs were distributed in five CRF01_AE strains and six subtype B strains.

**Table 4:** Baseline demographic characteristics of 11 participants with TAMs mutation

Sample code	Gender	Age	VL	CD4 counts	Occupation	WB date	ART regimen	Duration of ART	Transmission route	Genotype	NRTIs	NNRTIs
HB0041	Male	29	17,600	181	Worker	2010-7-9	3TC + d4T + NVP	26	Heterosexual	CRF01_AE	D67N, K70R, M184V, K219Q	A98G, K101E, G190A
HB0455	Male	39	51,200	85	Farmer	2010-1-22	3TC + AZT + EFV	34	Heterosexual	B	M41L, D67N, K70R, L74I, M184V, T215F, K219Q	K101E, V106I, V179E, G190S
HB0549	Female	43	50,800	170	Farmer	2011-4-8	3TC + AZT + NVP	20	Heterosexual	CRF01_AE	D67N, T69N, K70R, M184V, T215F, K219E	K103N, Y181C
HB0595	Male	41	195,000	7	Liberal professions	2009-1-9	3TC + AZT + NVP	15	Homosexual	CRF01_AE	D67N, K70R, M184V, T215F, K219E	Y188L
HB0711	Male	31	47,800	175	Jobless	2009-7-20	3TC + AZT + NVP	27	Heterosexual	CRF01_AE	D67N, T69N, K70R, V75M, M184V, T215I, K219Q	Y181C, H221Y
HB0829	Male	49	6360	187	Jobless	2007-5-31	3TC + AZT + NVP	32	Heterosexual	B	D67N, K70R, M184V, K219Q	G190A
HB0869	Male	36	26,500	15	Farmer	2010-6-28	3TC + AZT + NVP	33	Heterosexual	CRF01_AE	D67N, K70R, M184V, K219E	V90I, Y188W, F227L
HB1157	Male	50	10,600	184	Farmer	2003-7-16	3TC + AZT + NVP	10	Heterosexual	B	D67N, K70R, M184V, K219E	V90I, K103N, K238T
HB1160	Female	45	7030	9	Farmer	2003-4-1	3TC + TDF + LPV	133	Heterosexual	B	D67N, K70R, M184V, K219E	V90I, K103N, K238T
HB1161	Female	15	1580	136	Farmer	2004-2-19	3TC + AZT + NVP	80	Mother-to-child	B	D67N, K70R, M184V, K219E	V90I, K103N, K238T
HB1225	Female	71	46,400	59	Farmer	2001-12-14	3TC + d4T + NVP	84	Heterosexual	B	M41L, L74V, M184V, L210W, T215Y	K103N, V108I, Y181C, H221Y

*WB* western blot, *VL* viral load, *NRTIs* nucleoside reverse transcriptase inhibitors, *NNRTIs* non-nucleoside reverse transcriptase inhibitors

### The distribution of HIV-1 DR mutations among different genotypes

As shown in Table 5, there was no significant difference in the overall distribution of 15 main mutations in the RT coding region in CRF01_AE, subtype B, and CRF07_BC (*p* > 0.05). These 15 mutations largely resided in CRF01_AE and subtype B. Mutations T74S and M46L in the PI coding region were found in CRF01_AE. However, the distributions of M184 V/I (χ^2^ = 7.289, *p* < 0.05) and M41L (*p* < 0.05) were significantly different among CRF01_AE, subtype B, and CRF07_BC, respectively.

**Table 5:** The distribution of HIV-1 DR mutations in RTs coding region among different genotypes

Genotypes	Participants	Reverse transcriptase inhibitor mutations														
		M184V/I	M41L	T215Y/F/I/N	K70R	D67N	K65R	T69i	L74I/V	K103N/S/T	Y181C	V106A/M	Y188L/C	G190A/S	M230L/I	P225H
CRF01_AE	133	26	2	8	7	7	0	1	0	15	12	0	2	7	1	1
B	139	35	9	12	11	6	3	0	5	23	14	4	3	12	3	3
07-BC	45	3	0	0	0	0	0	0	0	2	0	1	0	0	0	0
C	4	1	0	0	0	0	1	0	0	0	1	0	0	0	0	0
URFs	2	0	0	0	0	0	0	0	0	0	1	0	0	0	0	0
CRF02-AG	2	0	0	0	0	0	0	0	0	0	0	0	0	0	0	0
08-BC	7	0	0	0	0	0	0	0	0	0	0	0	0	1	0	0
χ^2^ test		7.289	F	4.320	4.049	2.398	F	F	6.505	4.889	4.785	F	F	4.714	F	F
P		0.026	0.048	0.115	0.132	0.302	0.367	0.562	0.065	0.087	0.091	0.129	1.000	0.095	0.796	0.796

*F* Fisher’s exact test, *RTs* reverse transcriptase inhibitors

### Factors associated with HIV-1 drug resistance

As listed in Table 6, 14 potential risk factors were considered in the analysis of univariate logistic regression. Of these factors, VL, symptoms in the last 3 months, CD4 count, transmission route, duration of ART, and genotype were clearly related to HIV-1 DR (*p* < 0.05). To identify risk factors associated with HIV-1 DR, multivariable logistic regression analysis was implemented using stepwise selection. Five factors were found to be significantly associated with the progress of HIV-1 DR in participants experiencing treatment failure: transmission route (compared with sexual contact, blood: odds ratio (OR) 0.1, 95% confidence interval (CI) 0.03–0.24; mother-to-child: OR 1.2, 95% CI 0.3–4.3); CD4 count (>200 vs. ≤200 cells/µl: OR 0.2; 95% CI 0.1–0.5; *p* < 0.001); VL (compared with ≤5000 log copies/ml, 5001–9999: OR 1.7, 95% CI 0.6–5.0; ≥10,000: OR 4.9, 95% CI 2.0–12.0); duration of ART (compared with 0–12 months, 13–54 months: OR 1.7, 95% CI 0.8–3.8; ≥55 months: OR 3.8, 95% CI 1.4–10.1); and symptoms in the last 3 months (yes vs. no: OR 2.4; 95% CI 1.0–5.6; *p* < 0.05).

**Table 6:** Factors associated with HIV-1 DR among participants experiencing treatment failure

Factors	DR rate % (N)	Crude OR (95% CI)	p value	Adjusted OR (95% CI)	p value
Gender					
Male	51.6 (79)	1	0.913		
Female	52.5 (32)	1.034 (0.571, 1.872)			
Age (years)					
0–18	63.2 (12)	1	0.35		
19–49	52.5 (73)	0.646 (0.242, 1.725)			
50–	43.2 (26)	0.444 (0.143, 1.385)			
Marital status					
Married/cohabitation	48.5 (49)	1	0.473		
Unmarried	57.7 (41)	1.45 (0.787, 2.673)			
Divorced/widowed	50 (21)	1.016 (0.517, 2.18)			
Ethnicity					
Han	51 (106)	1	0.118		
Others	83.3 (5)	4.811 (0.553, 41.894)			
VL (copy/ml)					
≤5000	31.4 (16)	1	0.001	1	0.001
5001–9999	45.7 (16)	1.842 (0.756, 4.486)		1.673 (0.558, 5.01)	
≥10,000	61.7 (79)	3.527 (1.768, 7.035)		4.9 (1.996, 12.026)	
CD4 counts (cells/μl)					
≤200	68.4 (78)	1	0.001	1	0.001
>200	33 (33)	0.227 (0.128, 0.404)		0.219 (0.101, 0.474)	
WHO stages					
I	47.3 (71)	1	0.074		
II	76.5 (13)	3.616 (1.127, 11.6)			
III	52.9 (18)	1.252 (0.594, 2.639)			
IV	69.2 (9)	2.54 (0.739, 8.485)			
Symptoms^a^ in recent three months					
No	44 (66)	1	0.001	1	0.046
Yes	70.3 (45)	3.014 (1.162, 5.635)		2.386 (1.017, 5.594)	
Education level					
High school or less	51.3 (82)	1	0.755		
College and above	53.7 (29)	1.103 (0.595, 2.048)			
Duration between diagnosis and ART					
0–12	50.4 (66)	1	0.786		
13–54	57.7 (15)	1.343 (0.574, 3.142)			
55–	52.6 (30)	1.094 (0.587, 2.039)			
ART regimens					
3TC + AZT + NVP/EFV	52.8 (67)	1	0.621		
3TC + TDF + LPV/r	59.1 (13)	1.294 (0.516, 3.241)			
Others^b^	47.7 (31)	0.817 (0.449, 1.486)			
Duration of ART (months)					
6–12	36.8 (32)	1	0.001	1	0.025
13–54	57 (45)	2.275 (1.22, 4.242)		1.737 (0.801, 3.766)	
55–	70.8 (34)	4.174 (1.953, 8.923)		3.828 (1.445, 10.14)	
Transmission routes					
Sexual contact	59.9 (91)	1	0.001	1	0.001
Blood	19 (8)	0.158 (0.068, 0.364)		0.082 (0.028, 0.241)	
Mother-to-child	60 (12)	1.005 (0.388, 2.604)		1.156 (0.313, 4.268)	
Genotype					
B	55.4 (62)	1	0.035	1	0.484
CRF-1_AE	55.6 (40)	1.008 (0.556, 1.829)		0.765 (0.343, 1.708)	
Others^c^	30.0 (9)	0.346 (0.145, 0.821)		0.543 (0.195, 1.512)	

*OR* odds ratio, *DR* drug resisitance

^a^Herpes zoster, persistent diarrhea (>1 month), persistent/intermittent fever (>38 °C, >1 month), Brain lymphoma/B-cell non-Hodgkin’s lymphoma, tuberculosis or thrush

^b^3TC + d4T + NVP, 3TC + TDF + EFV, 3TC + D4T + EFV and 3TC + TDF + NVP were included

^c^CRF07_BC (4 cases), CRF08_BC (2 cases), CRF02_AG (1 case), C (1 case) and URF (1 case) were included

## Discussion

Following the phylogenetic analysis of HIV-1 *pol* sequences in the present study, we successfully identified two HIV-1 subtypes, four CRFs, and two URFs in 11 prefectures of Hebei Province, China. The HIV-1 genotype distribution was shown to be closely related to the route of transmission. Moreover, the prevalence of HIV-1 genotypes in this study differs significantly from that in Sichuan, Yunnan, and Xinjiang provinces, where IDUs are the common high risk group [10], suggesting that the prevalence of HIV-1 genotypes in different provinces of China reflects the geographical difference of HIV-1 high-risk populations.

Traditionally, HIV-1 subtype B was dominant in contaminated blood in the cities of Langfang and Xingtai [14, 22], and our work provides new evidence to support this. CRF01_AE strains in China were identified in IDUs for the first time in Yunnan [4]. Since the first CRF07_BC epidemic in 2002 [23], the prevalence of CRF07_BC has increased significantly, from 4.5% in 2002 to 13.6% in this study, and it has been identified in all transmission routes. From 1989 to 2013, a shift in transmission routes became apparent [15, 21], from which subtype B, CRF01_AE, and CRF07_BC spread out through sexual contact [21, 24] with an increasing diversity of high-risk behaviors and the growing size of the floating population. Currently, sexual transmission is the most common route of transmission in Hebei, accounting for 98.1% of HIV-1-positive cases in 2013 [21]. Subtype B, CRF01_AE, and CRF07_BC are the three main genotypes, and mainly circulate through sexual contact. The co-circulation of these three genotypes has resulted in the occurrence and spread of novel recombinant strains, as evidenced by the detection of recombinant strains CRF01_AE/B and CRF01_AE/BC in this study. To our knowledge, this is the first report of HIV-1 subtype specialty and DR mutations in Hebei.

In our work, the mutation classes of HIV-1 DR showed significant differences between ART-naïve controls and participants experiencing treatment failure. The prevalence of single, dual, and multiple mutations in participants experiencing treatment failure was significantly higher than in ART-naïve participants, which is consistent with previous findings in Yunnan [25]. The dual NRTI and NNRTI DR prevalence (29.4%) was highest, followed by that of NNRTIs (10.7%), NRTIs (4.2%), and PIs (2.8%) in participants experiencing treatment failure. However, in ART-naïve participants, the PI DR prevalence (2.5%) was higher than that of NRTIs (1.7%), NNRTIs (0.8%), and NRTIs and NNRTIs (0.8%), in contrast to an earlier report [26]. Our observed DR rate of 51.9% in participants experiencing treatment failure was higher than that seen in Henan Province (47.1–64.7%) [27, 28] and Switzerland (37–45%) [29], suggesting that the higher prevalence of HIV-1-resistant strains is closely related to the widespread use of antiviral drugs. This has occurred in China since 2003, after which time more HIV-1 drug-resistant variants were identified and have spread.

The prevalence of NNRTI mutations was higher than that of other mutations in this study, which might reflect the replicative fitness of the virus. For example, Y181C can increase HIV-1 subtype B replicative capacity [30]. Moreover, our study also revealed significant differences in the distributions of M184V/I and M41L mutations among three main genotypes, with M46L/V and T74S only found in CRF01_AE. The differences of HIV-1 mutation distribution in three main genotypes provide some clues of replicative fitness of the virus and renewal of the therapeutic regime. By contrast, the distributions of the remaining mutations were not significantly different among three main genotypes, suggesting that they are randomly distributed in these genotypes.

First-line antiretroviral drugs were included in all therapeutic regimens used in this study, and the prevalence of DR did not differ significantly among these regimens. Of all participants using therapeutic regimens containing lopinavir plus ritonavir (LPV/r), 59.1% showed resistance to partial PIs (Table 6). Factors associated with the high prevalence of HIV-1 DR among participants experiencing treatment failure are as follows: first, the higher VL (>5000 copies/ml) and lower CD4 counts (≤200 cells/µl) are closely related to the higher prevalence of HIV-1 DR. Moreover, compared with asymptomatic patients, HIV/AIDS patients who showed symptoms (Herpes zoster, tuberculosis or thrush, etc.) during the past 3 months had a higher prevalence of resistance to antiretroviral drugs. These phenomena provide new evidence for evaluating disease development and monitoring resistant strains using CD4 counts, VL, and clinical symptoms in the course of ART. Second, the duration between diagnosis and ART did not significantly affect the prevalence of DR. However, the ART duration was positively correlated with the prevalence of drug-resistant strains, indicating that following prolonged treatment, HIV-1 strains develop drug-selected mutations adapted to the antiretroviral drug. For example, TAMs resistant to all drugs listed in the HIV DR database were detected after a mean therapeutic duration of 42.8 months, and the long-term use of the first-line regimens may have played a role in the development of TAMs mutations. Additionally, individuals infected with HIV-1 transmitted drug resistance (TDR) strains derived from treated patients were resistant to related drugs. In this study, the most common mutations (M184V/I, K103N, Y181C, etc.) proved HIV-1 fitness to drugs, suggesting that HIV-1 resistant strains would spread out. Lu et al. [31] previously confirmed the existence of HIV-1 TDR strains among men who have sex with men in Hebei. Because the spread of HIV-1-resistant strains through sexual contacts is much faster than through other routes, timely behavioral intervention is particularly important.

## Conclusions

In contrast with our previous studies, we observed extensive HIV-1 genetic diversity in the present study. The high prevalence of HIV-1 DR in participants experiencing treatment failure of Hebei appears to have been induced by antiretroviral drug treatment after a longer duration of ART. Moreover, significant differences were observed in the distribution of mutations in different HIV-1 genotypes. Our study results suggest that we should formulate new prevention and control strategies according to geographic characteristics of HIV-1 genotype distribution and the epidemic characteristics of HIV-1-resistant strains in Hebei.

## Additional file

**Additional file 1.** Additional figures and tables.

## Abbreviations

HIV: human immunodeficiency virus
CRF: circulating recombinant form
SPSS: statistical package for the social sciences
IDU: intravenous drug injection
MTCT: mother-to-child transmission
ART: antiretroviral therapy
URF: unique recombinant form
PIs: protease inhibitors
NRTIs: nucleoside reverse transcriptase inhibitors
NNRTIs: non-nucleoside reverse transcriptase inhibitors
DR: drug resistance
ATV/r: atazanavir/r
NFV/r: nelfinavir plus ritonavir
LPV/r: lopinavir plus ritonavir
3TC: lamivudine
ABC: abacavir
AZT: zidovudine
D4T: stavudine
DDI: didanosine
FTC: emtricitabine
TDF: tenofovir
EFV: efavirenz
ETR: etravirine
NVP: nevirapine
