# Supplementary material for: HIV-1 drug-resistant mutations and related risk factors among HIV-1-positive individuals experiencing treatment failure in Hebei Province, China
Source: AIDS Res Ther. 2017 Jan 23;14:4. doi: 10.1186/s12981-017-0133-3 (PMC5260017; doi:10.1186/s12981-017-0133-3)
Supplement: Supplementary file 1 — Additional file 1. Additional figures and tables. [file 12981_2017_133_MOESM1_ESM.doc]

**HIV-1 drug-resistant mutations and related risk factors among antiretroviral therapy-failure HIV-1-positive individuals in Hebei Province, China**

Xinli Lu, Hongru Zhao, Yuqi Zhang, Wei Wang, Cuiying Zhao, Yan Li, Lin Ma, Ze Cui* and Suliang Chen*

Hebei Provincial Center for Disease Control and Prevention, 97 Huaian East Rd, Yuhua District, Shjiazhuang 050021, People’s Republic of China

XL: [lxlii2009@163.com](mailto:lxlii2009@163.com)

HZ: [lxli0124@163.com](mailto:lxli0124@163.com)

YZ: yqzhang688@163.com

WW: [lxlii2015@163.com](mailto:lxlii2015@163.com)

CZ: [zhaocuiying906@sina.com](mailto:zhaocuiying906@sina.com)

YL: hbstd666@163.com

LM: maixiangxiyu@126.com

ZC: [hbcdc888@163.com](mailto:hbcdc888@163.com)

SC: [hebeicdc2013@sina.com](mailto:xjkang@hbu.edu.cn)

*Corresponding authors: Ze Cui and Suliang Chen

Tel: +86-311-86573441

Fax: +86-311-86573371

**
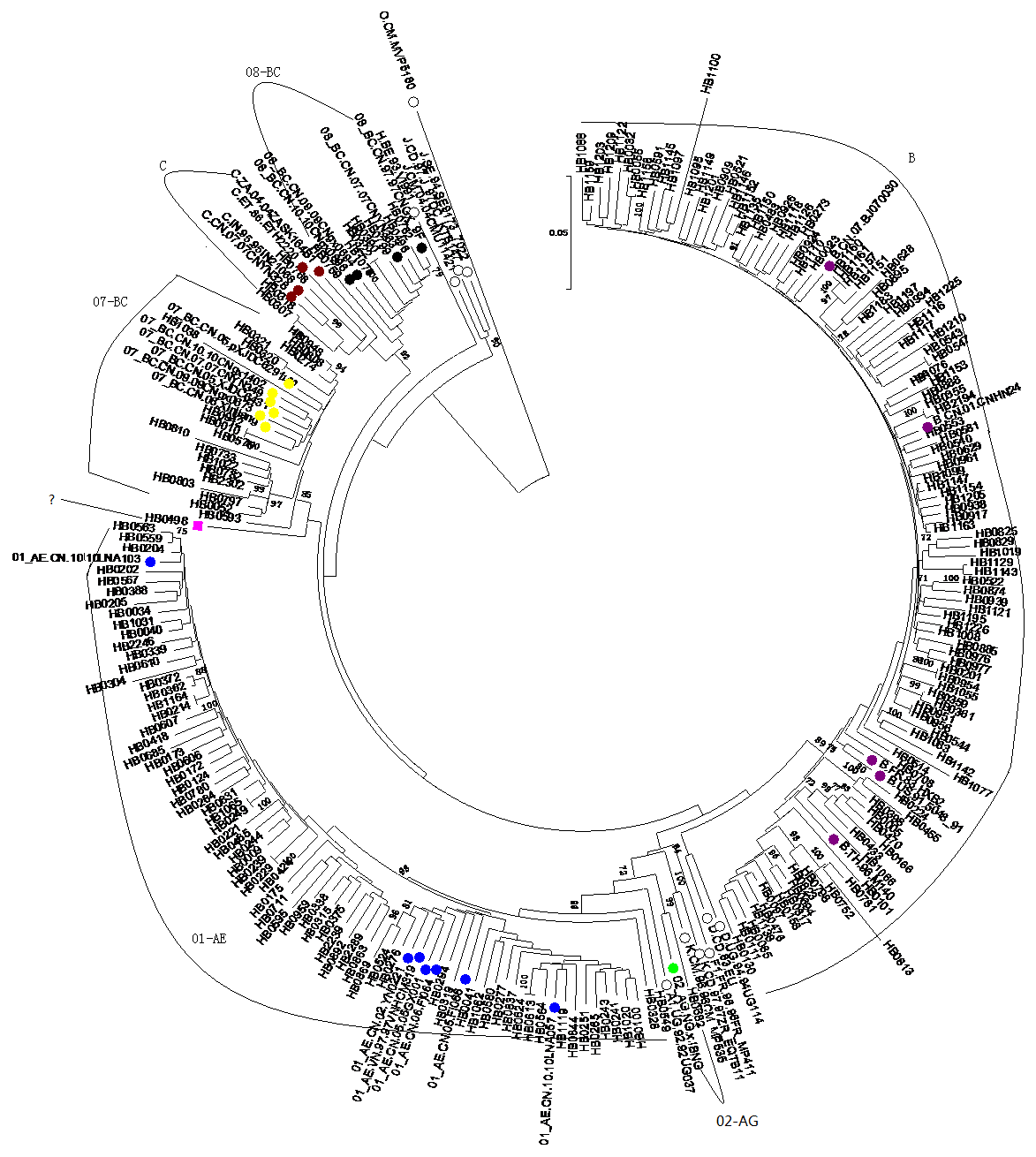
**

**Figure S1 The Neighbor-joining phylogenetic tree of partial *pol* gene sequences from ART-failure HIV-1 subjects**

Note: Each HIV-1 subtype reference sequence was labeled with different color. Black dot:CRF08_BC, brown dot:subtype C, yellow dot:CRF07_BC, blue dot:CRF01_AE, green dot:CRF02_AG, purple dot:subtype B, pink square block:URFs, white dot:other reference sequences

**
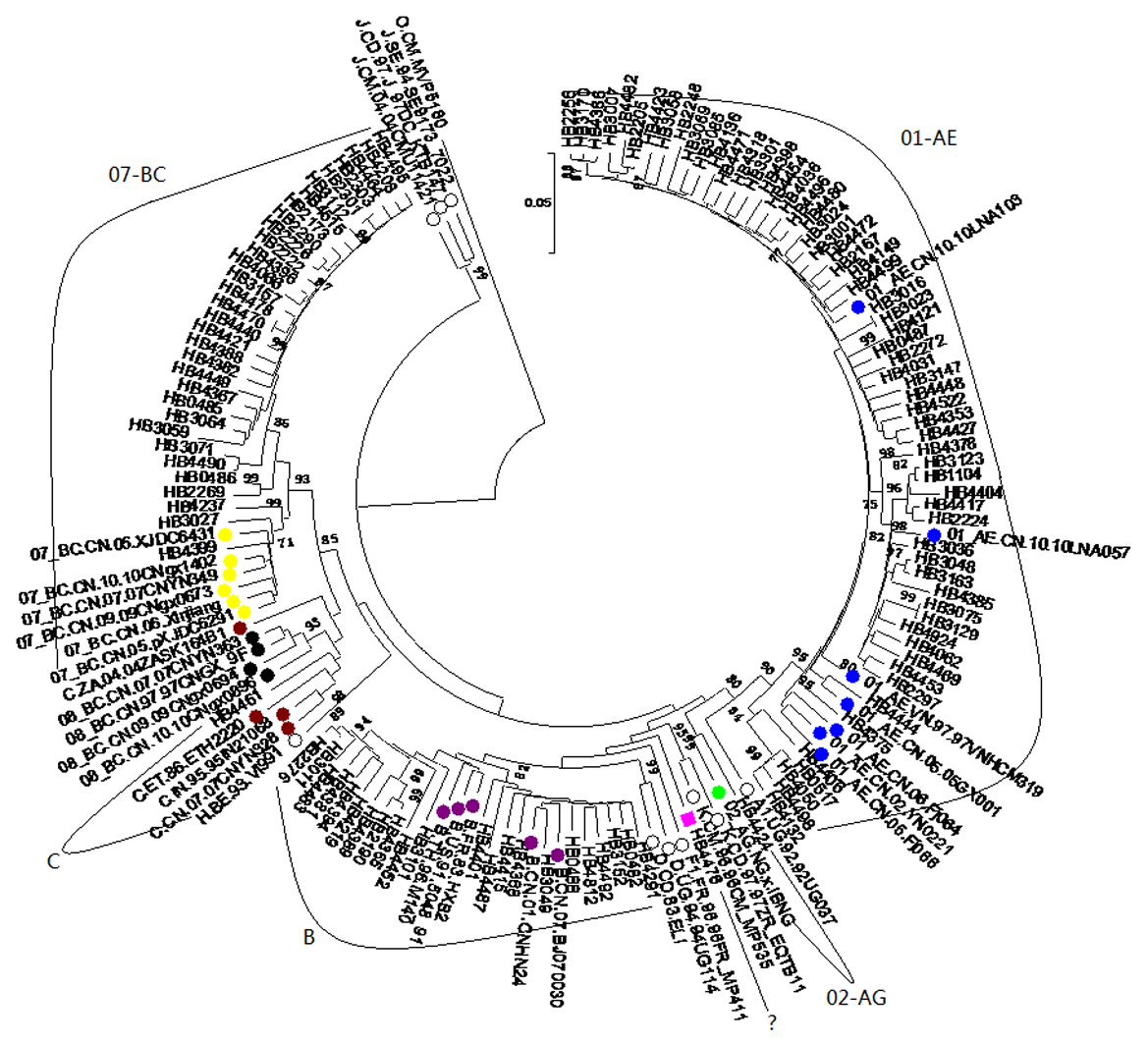
**

**Figure S2 The Neighbor-joining phylogenetic tree of partial *pol* gene sequences from newly diagnosed HIV-1 subjects**

Note: Each HIV-1 subtype reference sequence was labeled with different color. Black dot:CRF08_BC, brown dot:subtype C, yellow dot:CRF07_BC, blue dot:CRF01_AE, green dot:CRF02_AG, purple dot:subtype B, pink square block:URFs, white dot:other reference sequences


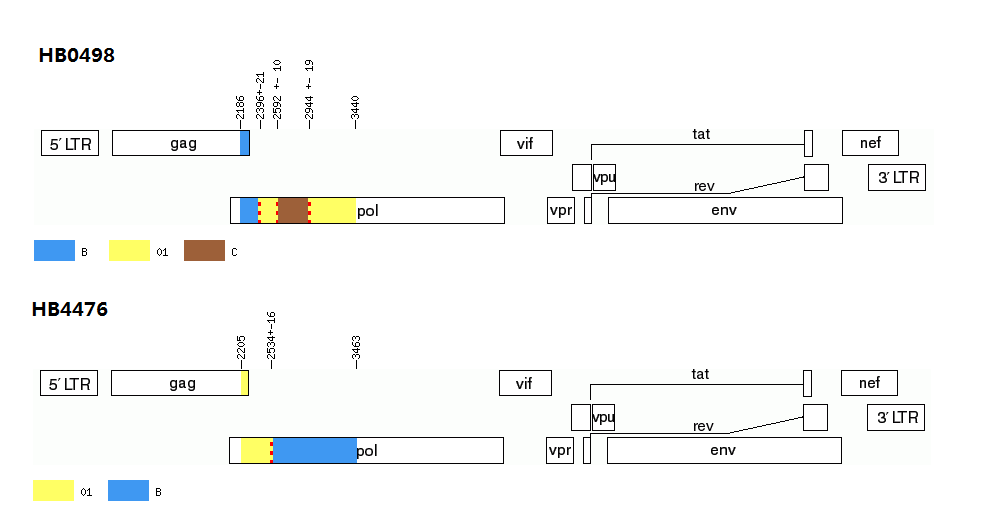


**Figure S3 Genomic maps of URFs(HE0498 and HB4476).** The mosaic maps were generated using jpHMM Program(<http://jphmm.gobics.de/submission_hiv.html>).

**
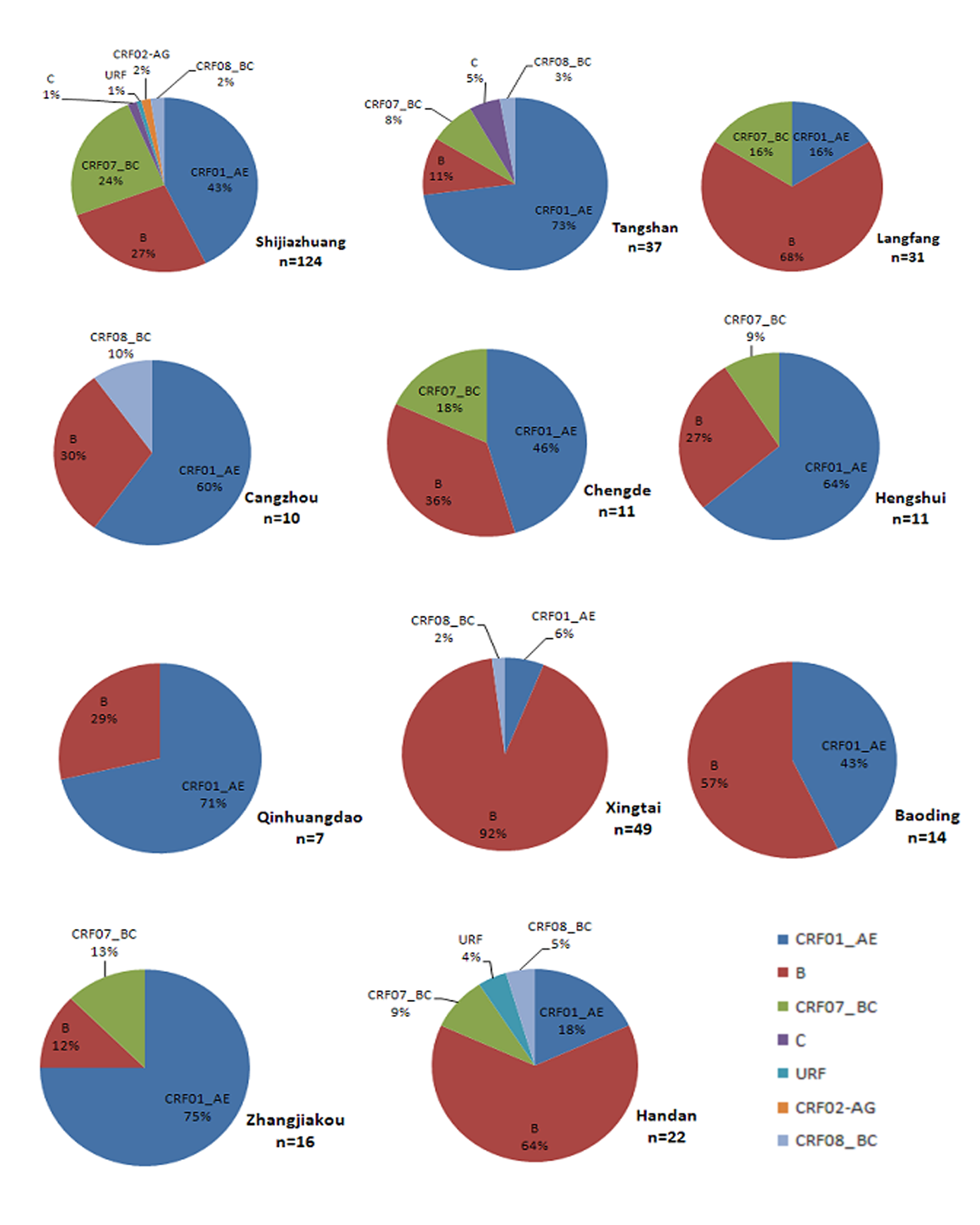
**

**Figure S4 Geographical distribution of HIV-1 genotypes in 11 prefectures of Hebei**

**Table S1 HIV-1 genotypes among newly diagnosed ART-naïve(n=118) and ART-failure subjects(n=214)**

| **Genotype** | **ART-naïve (%)** | **ART-failure(%)** | **Cumulative (%)** |
| --- | --- | --- | --- |
| CRF07_BC | 27 (22.9) | 18 (8.4) | 45 (13.6) |
| CRF08_BC | 0 (0.0) | 7 (5.9) | 7 (2.1) |
| B | 27 (22.9) | 112 (52.3) | 139(41.9) |
| CRF01_AE | 61 (51.7) | 72 (33.6) | 133(40.1) |
| CRF02_AG | 1(0.9) | 1 (0.5) | 2 (0.6) |
| URFs | 1(0.9) | 1（0.5） | 2(0.6) |
| C | 1(0.9) | 3（1.4） | 4(1.2) |

ART, antiretroviral therapy; URFs, unique recombinant forms

**Table S2 HIV-1 subtypes distribution in different transmission routes**

a，Fisher`s exact test result; MTCT, Mother-to-child; IDU, Intravenous drug injection; URFs, unique recombinant forms

| **Transmission routes** | **Subjects**  **(**%**)** | **CRF01_AE**  **(**%**)** | **B**  **(**%**)** | **CRF07-**  **BC(**%**)** | **C**  **(**%**)** | **URFs**  **(**%**)** | **CRF02-AG**  **(**%**)** | **CRF08-BC(**%**)** | **P** |
| --- | --- | --- | --- | --- | --- | --- | --- | --- | --- |
| Heterosexual | 98(100) | 28（28.6） | 47(48.0) | 11(11.2) | 3(3.1) | 1(1.0) | 2(2.0) | 6(6.1) | <0.001^a^ |
| Homosexual | 162(100) | 100(61.7) | 31(19.1) | 29(17.9) | 1(0.6) | 1(0.6) | 0(0.0) | 0(0.0) |  |
| MTCT | 18(100) | 0(0.0) | 16(88.9) | 1(5.6) | 0(0.0) | 0(0.0) | 0(0.0) | 1(5.6) |  |
| Blood recipient | 30(100) | 2(6.7) | 28(93.3) | 0(0.0) | 0(0.0) | 0(0.0) | 0(0.0) | 0(0.0) |  |
| Paid blood donor | 22(100) | 3(13.6) | 17(77.3) | 2(9.1) | 0(0.0) | 0(0.0) | 0(0.0) | 0(0.0) |  |
| IDU | 2(100) | 0(0.0) | 0(0.0) | 2(100) | 0(0.0) | 0(0.0) | 0(0.0) | 0(0.0) |  |
